# Clustering of charged colloidal particles in the microgravity environment of space

**DOI:** 10.1038/s41526-023-00280-5

**Published:** 2023-04-29

**Authors:** Hiroyuki Miki, Teruyoshi Ishigami, Junpei Yamanaka, Tohru Okuzono, Akiko Toyotama, Jitendra Mata, Honoka Komazawa, Yushi Takeda, Madoka Minami, Minori Fujita, Maho Doi, Tsunehiko Higuchi, Hiroshi Takase, Satoshi Adachi, Tetsuya Sakashita, Taro Shimaoka, Masae Nagai, Yuki Watanabe, Seijiro Fukuyama

**Affiliations:** 1 Graduate School of Pharmaceutical Sciences, Nagoya City University, 3-1 Tanabe, Mizuho, Nagoya, Japan; 2 Australian Centre for Neutron Scattering (ACNS), Australian Nuclear Science and Technology Organisation (ANSTO), Lucas Heights, NSW 2234 Australia; 3 Faculty of Pharmaceutical Sciences, Nagoya City University, 3-1 Tanabe, Mizuho, Nagoya, Japan; 4 Core Laboratory, Graduate School of Medical Sciences, Nagoya City University, 1 Kawasumi, Mizuho, Nagoya, Japan; 5 Japan Aerospace Exploration Agency (JAXA), 2-1-1 Sengen, Tsukuba, Japan; 6 Japan Space Forum (JSF), 3-2-1 Kandasurugadai, Chiyoda, Tokyo, Japan; 7 Advanced Engineering Services (AES) Co., Ltd., 1-6-1 Takezono, Tsukuba, Japan

**Keywords:** Colloids, Self-assembly

## Abstract

We conducted a charge–charge clustering experiment of positively and negatively charged colloidal particles in aqueous media under a microgravity environment at the International Space Station. A special setup was used to mix the colloid particles in microgravity and then these structures were immobilized in gel cured using ultraviolet (UV) light. The samples returned to the ground were observed by optical microscopy. The space sample of polystyrene particles with a specific gravity *ρ* (=1.05) close to the medium had an average association number of ~50% larger than the ground control and better structural symmetry. The effect of electrostatic interactions on the clustering was also confirmed for titania particles (*ρ* ~ 3), whose association structures were only possible in the microgravity environment without any sedimentation they generally suffer on the ground. This study suggests that even slight sedimentation and convection on the ground significantly affect the structure formation of colloids. Knowledge from this study will help us to develop a model which will be used to design photonic materials and better drugs.

## Introduction

Self-assembly of submicron to micron-sized particles has been observed in various phenomena in nature, including aggregation of unstable colloidal dispersions^1–4^ and association of proteins in living cells^5^. The formation of colloidal clusters, composed of a small number of colloidal particles, has been reported in numerous studies^6–11^. In particular, tetrahedral clusters are the building blocks of diamond lattices, which are photonic crystals capable of confining light^12–15^, and the conditions for their formation have been explored^16–21^. By using DNA-mediated nearest-neighbor interactions with pre-prepared superstructural building blocks, diamond lattices, and pyrochlore lattices have been obtained^18^. In colloidal cuboids dispersed in evaporating emulsion droplets, clusters are obtained that serve as “pre-assembled” mesoscale building blocks for larger-scale structures^22^. The colloidal self-assembly enables the construction of novel materials that are useful in the fields of photonics, optoelectronics and sensing, and even clinical diagnostics.

**Fig. 1:**
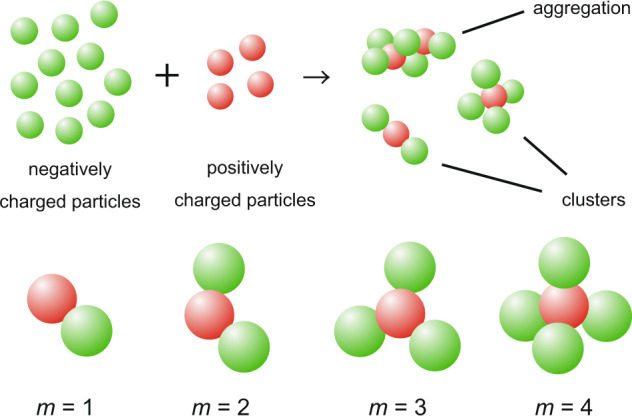
Colloidal clusters. The schematic of clustering and clusters of positively and negatively charged colloidal particles. *m* is the association number.

In this study, we examined the association of positively and negatively charged colloidal particles (hereafter referred to as p and n particles) by electrostatic attraction (Fig. 1). A schematic diagram of various clusters consisting of one p-particle and n-particles with an association number *m* (=1~4) is shown in Fig. 1. Tetrahedral clusters (*m* = 4) are of interest as building blocks of the diamond lattice, a photonic crystal with a perfect photonic band gap.

Colloidal particles with high refractive indexes (*n*_r_) are often required to construct materials with excellent optical properties. For example, a diamond lattice structure with particles with a refractive index *n*_r_ > 2 is required to achieve a perfect photonic band that reflects all incident light from all directions^16^. Generally, materials with high refractive indexes have, at the same time, high specific gravity *ρ*. For example, a titania (TiO_2_) particle with *n*_r_ = 2.4 has *ρ* > 3. On the ground, the structure formation of such heavy particles is often difficult due to gravitational settling effects. Assuming gravitational sedimentation with Stokes resistance *F* = 6*πηav* (*η*: viscosity of the medium, *a*: radius of the particle, *v*: settling velocity), *v* for 1 μm-sized titania particles at a temperature = 25 °C is 1.2 μm/s. This implies that the settling distance in one day is 5 cm. Microgravity environments, where sedimentation and convection are negligible, are ideal for experiments on the structure formation of micron-sized colloidal particles^23,24^.

A number of microgravity experiments were conducted on colloidal crystallization in rigid sphere dispersions. Here colloidal crystallization is a phenomenon in which particles are regularly arranged in a liquid, and particles in a crystalline structure are not in contact with each other. Microgravity experiments using the space shuttle revealed that particle sedimentation significantly affects nucleation and growth processes^24^. It has also been reported that dendritic growth is the dominant crystal growth mode under microgravity^25^. These have led to a review of the crystallization kinetics of hard spheres, and models have been devised that incorporate the interaction of diffusion fields in crystals^26,27^. We have also reported that the nucleation rate also decreases in the crystallization of charged colloids under microgravity obtained by parabolic flight^28^. However, the formation of clusters, in which particles are in contact with each other, has not been reported so far.

We conducted a clustering experiment of positively and negatively charged colloidal particles in aqueous media on the Japanese Experiment Module (JEM) “Kibo” of the International Space Station (ISS) in 2020^29^. p- and n- particles of about 1 µm were mixed in the microgravity environment of the ISS to form clusters. The main driving forces for clustering were electrostatic interactions and van der Waals (vdW) attraction between particles. Polystyrene (PS) particles, which have almost the same *ρ* (=1.05) as water, and titania particles with high refractive index and high specific gravity (*ρ* ~ 3) were used as colloidal particles. In this paper, we report the results of the analysis of colloidal clusters generated in space.

## Results and discussion

### Overview of our space experiments

**Fig. 2:**
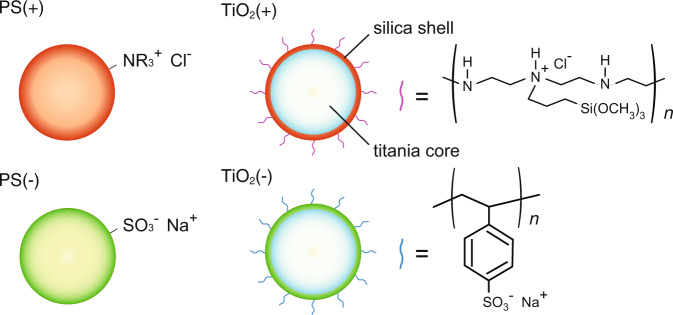
Colloidal particles. An illustration of the particles used in the space experiments.

Here, we briefly describe our space experiment on the ISS. Additional information is provided in Supplementary Note 1. The colloidal particles we used are illustrated in Fig. 2. Their properties are shown in Table 1a. Negatively charged fluorescent PS particles were obtained from Thermo Scientific (Massachusetts, U.S.A.). Other particles were synthesized by methods described in Supplementary Note 2. The titania particles were coated with a silica layer on their surfaces, and a polyelectrolyte was introduced to the silica surface to provide them with electric charges. p- and n-particles were stained with red (rhodamine B isothiocyanate) and green (fluorescein isothiocyanate) fluorescent dyes for identification. Samples were prepared with various sodium chloride NaCl concentrations, [NaCl], to adjust the electrostatic interaction magnitude.

**Table 1:** Samples used in the space experiments.

a Properties of the colloidal particles		
Particle	*d*	ζ
	(nm)	(mV)
PS(+)	789±22	38.7
PS(-)	1025±36	-50.1
TiO2(+)	957±33	48.7
TiO2(-)1	907±35	-56.2
TiO2(-)2	1016±38	-50.4
TiO2(-)3	1004±32	-44.5
TiO2(-)4	1016±38	-36.1

b Composition of the samples						
Sample No.	p	n	*C*p	*C*n	[NaCl]	[HCl]
			(10^-3^ vol%)	(10^-3^ vol%)	(mM)	(mM)
1	PS(+)	PS(-)	2	10	0	10
2	PS(+)	PS(-)	2	10	50	10
3	PS(+)	PS(-)	2	10	100	10
4	PS(+)	PS(-)	2	10	200	10
9	TiO_2_(+)	TiO_2_(-)1	48	2	0	0
14	TiO_2_(+)	TiO_2_(-)2	48	2	0	0
19	TiO_2_(+)	TiO_2_(-)3	48	2	0	0
23	TiO_2_(+)	TiO_2_(-)4	48	2	0	0
24	TiO_2_(+)	TiO_2_(-)4	48	2	50	0
25	TiO_2_(+)	TiO_2_(-)4	48	2	100	0
26	TiO_2_(+)	TiO_2_(-)4	48	2	200	0

The composition of the space samples is compiled in Table 1b. Our previous study^30^ and the ground-based studies have shown that aggregates containing multiple particles with the same charge as the central particle are formed at high particle concentrations and small concentration ratios. The following experimental conditions were chosen to obtain isolated clusters: (i) the concentration of p- and n-particles must be sufficiently dilute (less than about 0.1 vol% in total), and (ii) the composition must be highly asymmetric, with a number concentration ratio of twenty or higher. UV curable gelation reagents^31,32^ had been dissolved in the colloidal samples. They were composed of 4.5v% dimethylacrylamide (gel monomer), 1.5 mg/mL methylene-bis-acrylamide (cross-linker), and 0.1 mg/mL VA-086 (photo-induced polymerization initiator). They undergo radical polymerization upon UV irradiation, forming a three-dimensional network structure in water, and the medium becomes a polymer gel.

**Fig. 3:**
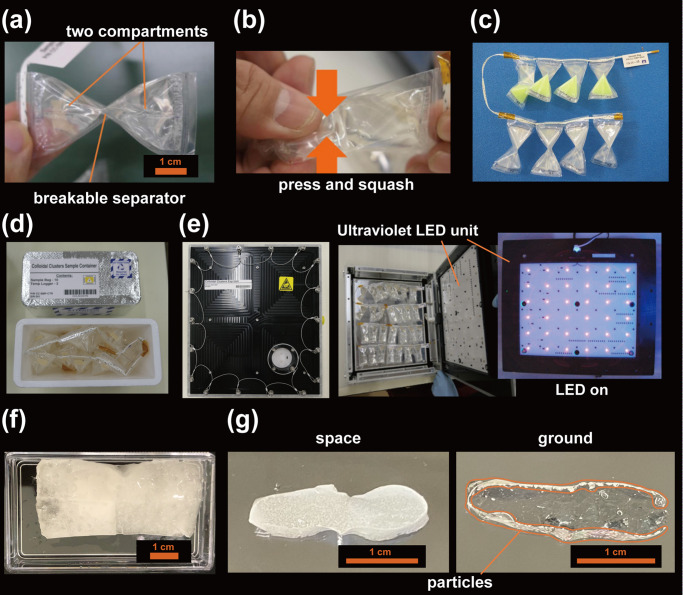
Experimental setup and samples used in the space experiment. **a** Sample bag, consisting of two tetra-pack compartments connected via a breakable separator. **b** The solution in the two chambers is mixed by squeezing the bag. **c** An example of a sample used in a space experiment. The sample is tied with string for proper handling in microgravity. **d** Sample bag in a shipping container. **e** The UV irradiation system. The bag is placed in the apparatus and the sample is immobilized by UV irradiation. The sample is dissolved in a UV-curing gelator. **f** Sample (Titania #23) returned to the ground. The sample was removed from the bag. **g** Cross section of the gel-fixed space sample and the ground sample. The space sample is uniformly white, while the particles in the ground sample are localized near the gel surface.

Figure 3a is an overview of our experimental apparatus. Two polymer bags of tetra-pack shapes with a volume of 3 mL are connected by a breakable seal. The connection is broken by crushing the bag to mix the liquids in the bags (Fig. 3b). The two bags were filled with dilute aqueous dispersions of p- and n-particles, respectively. Figure 3c shows an example of the sample used in the space experiment; a string connects the bags to prevent the sample from scattering under microgravity in the ISS. Figure 3d is the sample bag in the shipping container, which was transported to the ISS using this container. Figure 3e is a metal UV irradiation device. Sample bags (30 pieces) were fixed inside the apparatus, and the samples were gelled by UV irradiation with a UV-LED (365 nm, 9.0 W) installed behind the box.

The space experiment was carried out approximately eight months after the sample preparation. The space crew mixed the two liquids at ISS by pushing and squashing alternately 100 times to have homogeneity within the samples. Preliminary studies on the ground showed that it takes approximately two days to cluster the particles to reach nearly equilibrium. In the space experiment, the sample was left in microgravity for two days and gelled by UV irradiation. More details are given in Supplementary Note 1 and 3.

Figure 3f shows the appearance of the space sample (TiO_2_ # 23) taken out of the bag after returning to the ground. All of the space samples were successfully immobilized by the gel. The elastic modulus of gel *E* was ~25 kPa. This value was about 70% of the value when the samples were gelled immediately after preparations (*E* ~ 35 kPa), but it was large enough for the sample handling. The samples were then cut with a surgical scalpel, and the cross sections were observed by optical microscopy. Figure 3g compares the cross sections of the space samples and ground control samples prepared under a pseudo-microgravity obtained by the clinostat. No distinct supernatant was observed in the space sample, though the ground control sample had significant particle precipitation and the center of the sample was transparent.

A gelled space experimental sample portion was also sent to the Australian Nuclear Science and Technology Organisation (ANSTO) for small- and ultra-small-angle neutron scattering experiments to obtain information on the number of associations and aggregation structure.

### Ground control experiments

On the sample preparation, two samples having the same composition were made. One was used for the space experiment and the other as a ground control sample. The ground control samples were stored at 4 °C in JAXA Space Center (Tsukuba, Japan) for approximately eight months, as was the case of the space experiment. The control experiments were carried out in August 2020.

Some of the bags of PS and titania samples were opened without gelation and analyzed to examine changes in characteristics of samples during the storage. For the remaining samples, clustering experiments were conducted. On the ground, the sedimentation of the particles in two days is significant, particularly for titania particles. The settling distances estimated from the Stokes law in two days are 2.6 mm and 10 cm for PS and titania, comparable to and much greater than the inner dimension of the sample bag. On the ground, after the p- and n-particles were mixed by squashing the bags in the same manner in the space experiment and by rotating them gently, they were placed under pseudo-microgravity for two days. For PS and titania samples, we used a rotator (3 rpm) and a clinostat device (10 rpm). The samples were then immediately gelled by UV irradiation.

### Association number of PS clusters

**Fig. 4:**
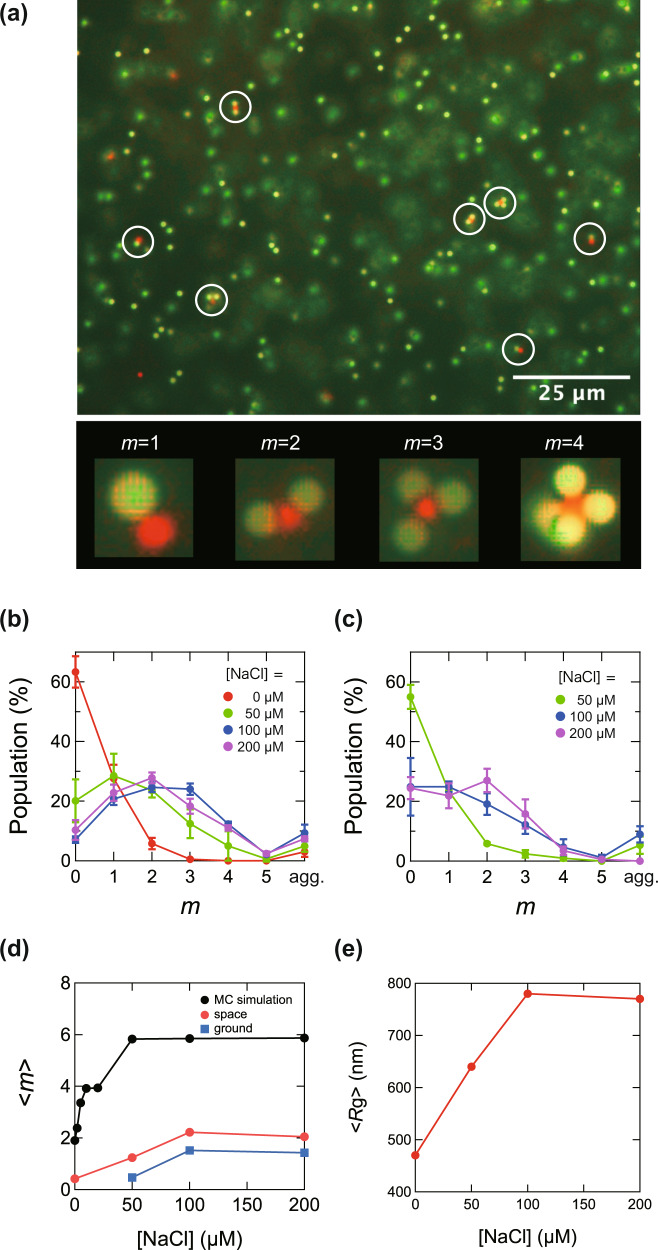
Microscopic images of PS clusters and distribution of association number. **a** Fluorescence micrographs of polystyrene (PS) clusters from the space experiment and magnified images of *m* = 1 to *m* = 4 clusters. **b**, **c** Distribution of the number of clusters *m* at various NaCl concentrations in the (**b**) space experiment and (**c**) ground experiment. **d** The plot of average cluster number <*m*> vs. [NaCl]. MC simulation results are also shown. **e** The plot of the average radius of inertia <*R*_g_> vs. [NaCl] for clusters obtained by neutron scattering.

Figure 4a shows a micrograph of sample #2 ([NaCl] = 50 μM). Because the PS samples have a large excess of n-particles, their isolated clusters are centered on p-particles and surrounded by n-particles. As shown in Fig. 4a, clusters with various association numbers *m* were identified in the space sample. Figure 4b shows the distribution of *m* obtained from the results of about 1000 clusters in samples #1-#4 ([NaCl] = 0, 50, 100, and 200 μM), respectively. With increasing [NaCl], the electrostatic shielding effect becomes stronger, and the Coulomb force between particles becomes weaker. Note that a small number of clusters containing two or more p-particles were also present (<6% of the entire p-particles); they were classified as “aggregates” (Notated as “agg.” In the horizontal axis).

Figure 4c shows the distribution of *m* for the ground control. Because the sample at [NaCl] = 0 μM was used for particle characterization without gelation, clustering experiments were carried out for samples at [NaCl] = 50, 100, and 200 μM. Figure 4d shows a plot of the average association number <*m*> versus [NaCl]. In both the space and ground experiments, <*m*> increased with increasing [NaCl], reaching a maximum at [NaCl] = 100 μM.

The mechanism of the clustering will be discussed in a separate subsection. As seen from Eq. (2), the electrostatic interaction between the particles is shielded by ions and decreases with increasing salt concentration *C*_s_. We note that in this paper *C*_s_ is the sum of the added NaCl concentration and the impurity ion concentration, and will be distinguished from [NaCl] in this paper *C*_s_ ∝ *κ*^2^, where *κ* is the Debye parameter.

**Fig. 5:**
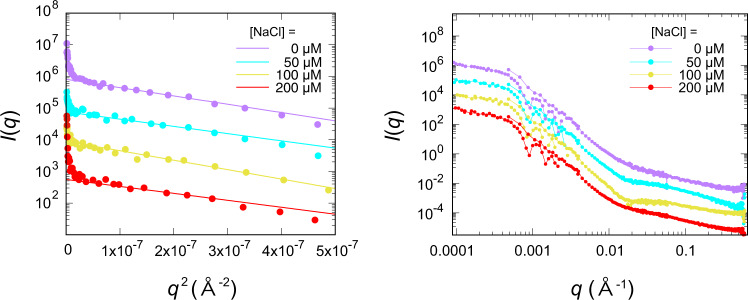
SANS-USANS scattering profiles (left), and Guinier plot (right) for PS clusters in the space sample for four [NaCl] values. The curves are vertically shifted and superimposed for clarity. In the Guinier region, scattering intensity *I*(*q*) is proportional to exp(-*R*_g_^2^*q*^2^),where *R*_g_ is the radius of gyration of the clusters, and *q* is the scattering vector. *R*_g_ is obtained as the slope of the plot of log *I*(*q*) versus *q*^2^.

Figure 4e shows the relationship between the [NaCl] and mean radius of gyration <*R*_g_> of the clusters obtained by the Guinier plot from ultra-small angle neutron scattering. The scattering curves are given in Fig. 5. The [NaCl] dependence trends of <*R*_g_> and <*m*> are in good agreement. Thus, the statistical scattering measurements corroborated the results obtained from microscopic observations.

### Structural symmetry of PS clusters

**Fig. 6:**
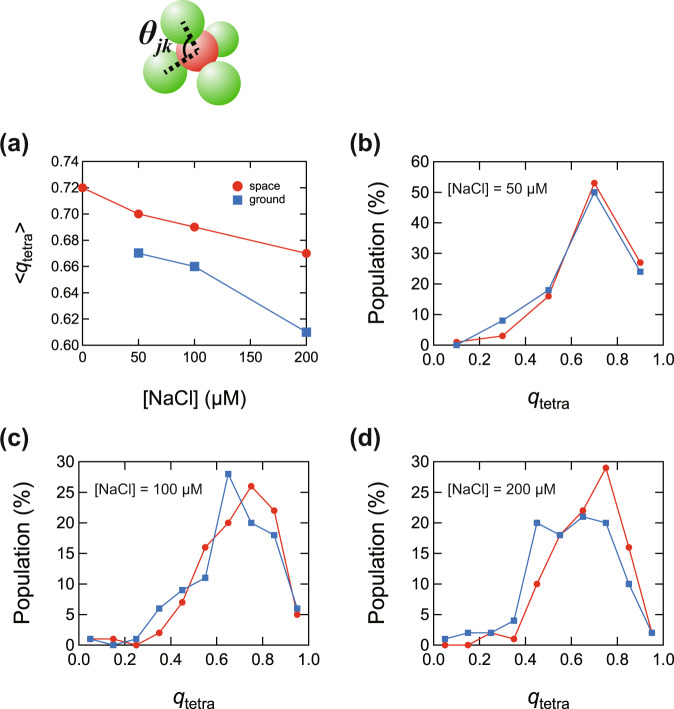
Comparison of the bond orientational order parameter *q*_tetra_ for PS particle tetrahedra produced by the space and ground experiments. **a** Plot of mean <*q*_tetra_ > versus [NaCl], **b**–**d** distribution of *q*_tetra_ at [NaCl] = 50 μM, 100 μM, and 200 μM.

We then examined the structural symmetry of tetrahedral (*m* = 4) PS clusters to examine the effects of microgravity in more detail. The relative positions of each particle in the tetrahedral clusters were determined by confocal microscopy. The structural symmetry of the tetrahedral clusters can be quantified by the bond orientational order parameter *q*_tetra_^33,34^, defined as

1 $${q}_{{\rm{tetra}}}=1-\frac{3}{8}\mathop{\sum}\limits_{j=1}^{3}\mathop{\sum}\limits_{k=j+1}^{4}{\left({\rm{cos }}{\theta }_{{jk}}+\frac{1}{3}\right)}^{2},$$

where *θ*_*jk*_ is the angle between the bonds of particles *j* and *k* (illustrated in Fig. 6). For a perfect tetrahedron, *q*_tetra_ = 1. Figure 6a is the mean *q*_tetra_ value, <*q*_tetra_>, for tetrahedrons of the PS particles at various [NaCl] values in the space and ground experiments. Figure 6b–d depicts the *q*_tetra_ distribution for the space and ground experiments at the three [NaCl] values. In the entire [NaCl] regions, the samples obtained from the space experiment had larger <*q*_tetra_>, that is, better symmetry, than the ground samples.

The same symmetry evaluation was performed for *m* = 2 and *m* = 3 clusters. The better symmetry of the space sample versus the ground control depends on the salinity, which was not as clear as in the case of *m* = 4. See Supplementary Note 5 for details. Further investigation is planned in conjunction with computer simulation results.

We note that the image of PS particles by high-resolution TEM shows the presence of roughness of several 10 nm on their surfaces. It is likely that the symmetry of the clusters is affected by frictional forces due to the non-smooth surface or directional entropy forces^35^. Elucidation of the impact of these effects is an important future issue.

### Clusters of titania particles

Cluster formation of titania particle systems were investigated by analyzing space samples, which is difficult to study on the ground due to gravitational sedimentation. In the titania sample, the central particle of the isolated cluster is an n-particle because p-particles are present in significant excess. The gel-immobilized space samples were uniform and no sedimentation effects were observed (Fig. 3g).

However, two unexpected things happened in the titania space experiment. One was the formation of numerous aggregates of more than 1000 particles in the sample. The other was the fluorescent dye discoloration; p-particles were stained with a red fluorescent pigment, but many of them turned red-green in color. Both of these issues were probably due to the degradation of the particles over time. Similar aggregation and fading of fluorescence were observed for the ground control sample, which was prepared and stored at the same time as the space experiment sample. The surface of the synthesized titania particles was coated with a silica layer, but analysis of the silicic acid concentration in the samples showed that some of it were leached out. The amount of leached silica in the space and ground samples was similar at 25% and 16%, respectively. Details of the degradation are described in Supplementary Note 6.

**Fig. 7:**
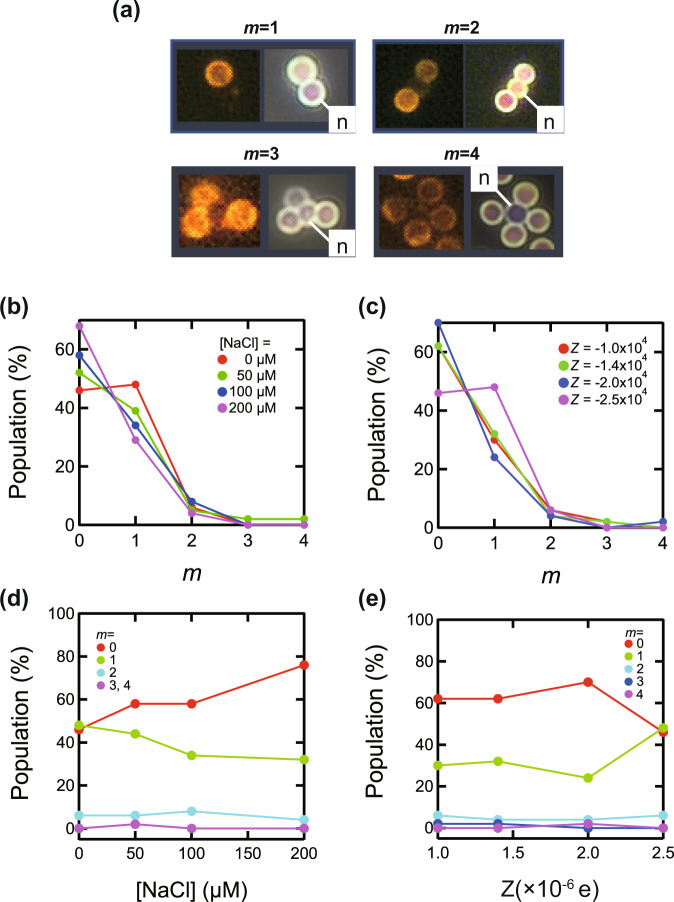
Microscopic images of titania clusters and distribution of association number. **a** Magnified images of titania-based clusters from *m* = 1 to *m* = 4 (left and right images with red fluorescence only detected and white light source, respectively). Distribution of the number of clusters *m* in the titania-based sample. **b** [NaCl]-dependence and **c** *Z*-dependence. **d** [NaCl] dependence and **e** *Z*-dependence of the proportion of *m* = 0 to *m* = 4 clusters.

On the other hand, various isolated clusters were also observed, as shown in Fig. 7a. Because the n-particles were stained green and did not exhibit red coloration, the p- and n- particles were distinguished by the presence of a red-colored component. Images of red fluorescence (left) and white light (right) are shown for each cluster in Fig. 7a. p-particles are observed in both pictures, while n-particles are observed only in white light.

Figure 7b shows distributions of *m* at various [NaCl] values. The population of *m* = 1 clusters was more significant at lower [NaCl], in accordance with the stronger electrostatic attraction between n- and p-particles. Figure 7c shows the charge number *Z* dependence, confirming that the number of *m* = 1 clusters became more significant with increasing *Z*. Note that *m* = 4 clusters were observed at [NaCl]=50 μM. In Fig. 7d, e, we also present the population plot of various clusters plotted against [NaCl] and *Z*, respectively. Thus, the space experiment confirmed the effect of electrostatic interaction on the titania particles’ clusters, which is difficult to verify on the ground.

### The aggregation structure of titania particles

**Fig. 8:**
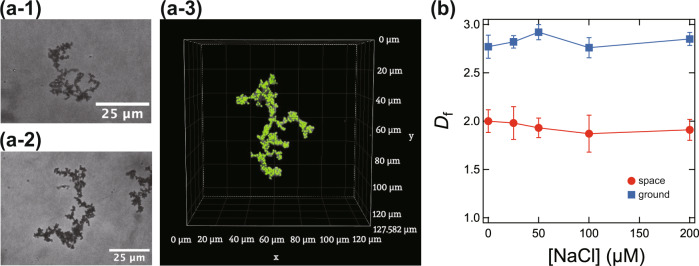
Microscopic images of aggregates formed in the titania-based space experimental samples. (**a-1**), (**a-2**) inverted microscope images, and (**a-3**) the confocal laser microscope image. **b** Fractal dimension *D*_f_ of the aggregates obtained for samples having various [NaCl] values. Red and blue symbols represent results for space and ground control samples, respectively.

Figure 8a-1–a-3 shows microscopic images of large aggregates of titania particles. These aggregates are made almost exclusively of p-particles.

The fractal dimension of the aggregates was determined from the microscopic images^36–40^. Figure 8b shows the fractal dimension *D*_f_ determined for samples with various [NaCl] values (red symbols). *D*_f_ was largely independent of [NaCl] and ranged between reaction-limited aggregation (~2.1) and diffusion-limited aggregation (~1.7). We note that the titania particles form dense aggregates of approximately *D*_f_ = 3 on the ground due to significant sedimentation (shown by blue symbols). The bulky aggregates with *D*_f_ = 2 observed in a microgravity environment again indicate the effect of microgravity on the assembly of titania particles. Thus, the significant influence of microgravity on the aggregate formation of high specific gravity particles was verified.

### Discussion on clustering of charged colloids

Here we discuss the clustering of the oppositely charged colloids in terms of interparticle interaction. The main driving forces for the clustering of charged colloidal particles are electrostatic interactions and vdW attraction between particles. The former is often discussed using the Yukawa-type two-body interaction potential *U*_Yukawa_(*r*), where *r* is the distance between the centers of the two particles, as

2 $${U}_{{\rm{Yukawa}}}\left(r\right)=\frac{{\rm{exp }}({\kappa a}_{1})}{1+\kappa {a}_{1}}\frac{{\rm{exp }}({\kappa a}_{2})}{1+\kappa {a}_{2}}\frac{{Z}_{1}{Z}_{2}{e}^{2}}{4\pi \varepsilon }\frac{{\rm{exp }}(-\kappa r)}{r},$$

where *a*_1_ and *a*_2_ are the radius of the two particles, *Z*_1_ and *Z*_2_ are the signed charge numbers, *ε* is the dielectric constant of the medium, and *e* is the elementary charge. *κ* is the Debye parameter, where *i* is the type of the *i*-th ion in the medium, and *c*_*i*_ and *z*_*i*_ are their concentrations and valences are given by

3 $${\kappa }^{2}=\frac{{e}^{2}}{\varepsilon {k}_{\text{B}}T}\sum {c}_{i}{z}_{i}^{2}.$$

Here *k*_B_ is the Boltzmann constant, and *T* is temperature. It can be seen that the higher the ion concentration, the more pronounced the electrostatic interaction is shielded. The vdW potential *U*_vdW_(*r*) acting between the two particles^41,42^ is given by

4 $${U}_{{\rm{vdW}}}(r)={Af}(r),$$

5 $$f\left(r\right)=-\frac{1}{6}\left(\frac{2{a}_{1}{a}_{2}}{{r}^{2}-{\left({a}_{1}{\,+\,a}_{2}\right)}^{2}}+\frac{2{a}_{1}{a}_{2}}{{r}^{2}-{\left({a}_{1}{-a}_{2}\right)}^{2}}+{\rm{ln}}\frac{{r}^{2}-{\left({a}_{1}{\,+\,a}_{2}\right)}^{2}}{{r}^{2}-{\left({a}_{1}{\,-\,a}_{2}\right)}^{2}}\right),$$

Here *A* is the Hamaker constant, which according to Lifshitz theory^41^, is given as *A* = *A*_ν=0_ + *A*_ν>0_ (ν is the frequency of electromagnetic waves), and

6 $${A}_{\nu =0}=\frac{3}{4}{k}_{{\rm{B}}}T\left(\frac{{\varepsilon }_{r1}-{\varepsilon }_{r3}}{{\varepsilon }_{r1}+{\varepsilon }_{r3}}\right)\left(\frac{{\varepsilon }_{r2}-{\varepsilon }_{r3}}{{\varepsilon }_{r2}+{\varepsilon }_{r3}}\right),$$

7 $${A}_{\nu > 0}=\frac{3h{\nu }_{e}}{8\sqrt{2}}\frac{({n}_{1}^{2}-{n}_{3}^{2})({n}_{2}^{2}-{n}_{3}^{2})}{{({n}_{1}^{2}+{n}_{3}^{2})}^{\frac{1}{2}}{({n}_{2}^{2}+{n}_{3}^{2})}^{\frac{1}{2}}\left[{({n}_{1}^{2}+{n}_{3}^{2})}^{\frac{1}{2}}{+({n}_{2}^{2}+{n}_{3}^{2})}^{\frac{1}{2}}\right]}.$$

Here *ε*_r*i*_ and *n*_r*i*_ are the particle’s relative permittivity and refractive index (*i* = 1, 2) and the medium (*i* = 3); *h* is the Planck constant, and *ν*_e_ is the principal absorption frequency of the material (~ 3 × 10^15^/s).

*f*’(*r*) in Eq. (4) is determined by the size of the two particles, while the *A* value depends on the difference between the permittivity and the refractive index of the medium and particles. For example, the *A* between two polystyrene particles (*n*_r_ = 1.59, *ε*_r_ = 2.5)^43^ interacting across water is 4.80 *k*_B_*T*, and 49.4 *k*_B_*T* for titania (*n*_r_ ~ 3, *ε*_r_ = 48)^43^ particles. Computer simulations used the two-body potential to study the relationship between the number of associations and the charge number of particles.

The attraction between p- and n-particles was stronger at lower *C*_s_. The smaller <*m*> at lower *C*_s_ can be attributed to stronger electrostatic repulsion between n particles, preventing additional attachments of n particles to the clusters. As *C*_s_ increased, reduction of the electrostatic repulsion between n particles causes increased in <*m* > . However, on further increase in *C*_s_, the attraction between the n- and p- particles became weaker, resulting in the reduction of <*m*>, as observed in the experiments. This trend is consistent with the results of the Monte Carlo simulation. In the polystyrene colloids, the number of associations and symmetry of clusters was superior to the space experiments. These results suggest that slight sedimentation and convection effect on the ground may affect the association of micrometer-order colloidal particles.

### Aggregation structure of titania

Here we discuss the mechanism of large aggregate formation in titania colloids. The titania particles we used were covered with silica shell layer as illustrated in Fig. 2. The determination of silica concentration in the medium of the space sample showed that ~25% of the silicates in the shell are dissolved in the medium. The dissolution of the silica shell may have two effects on the interparticle interactions. (i) The electrostatic repulsion is reduced because the polyelectrolyte on the silica surface is also removed, resulting in reduced *Z* values. (ii) The refractive index of the titania layer is about twice that of silica, and the thinner the silica layer, the stronger the vdW attraction between particles. The *Z* value of the ground control sample was reduced by about 28% from the initial values, according to (i). In addition, since the silica surface is negatively charged, partial desorption of the positive macromolecules of the p-particles could have resulted in a negatively charged patch region of some of the particles, which could have agglomerated due to Coulomb attraction between the positively charged portions of the other particles. We assume that the large aggregates observed were produced for these reasons. Details follow.

### Electrostatic interaction

We calculated electrostatic and vdW interactions for positively charged TiO_2_-SiO_2_ core-shell particles (TiO_2_ (+)), to investigate the formation mechanism of macroscopic aggregates observed for titania samples. The silica layer’s thickness and the medium’s salt concentration were parameters.

**Fig. 9:**
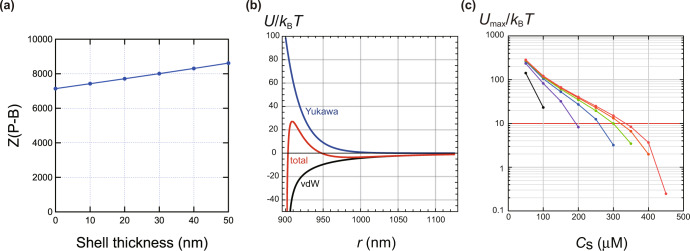
Estimation of the effect of silica coat thickness on the number of charges and particle-particle interactions in titania particles. **a** Number of particle charges calculated assuming various SiO_2_ shell layer thicknesses. **b** Interaction potential between TiO_2_-SiO_2_ core-shell particles (Shell thickness 20 nm, *C*_s_ = 200 µM). **c** Maximum values of the interaction potential between TiO_2_-SiO_2_ core-shell particles (From top, shell thickness = 50, 40, 30, 20, 10, and 0 nm).

The TiO_2_ core size of the sample was estimated to be 860 nm from SEM observations, and the thickness of the silica layer was 50 nm when the sample is filled. Henry’s equation determined the zeta potential from the measured electrophoretic mobility of the ground control particles without gel fixation, and the surface charge number *Z*(P-B) of the particles was calculated using the Poisson-Boltzmann equation *Z*(P-B) depending on the particle size, which in turn depends on the shell thickness. The calculation results are shown in Fig. 9a.

Yukawa potentials were calculated for various salt concentrations according to Eq. (2).

### vdW interaction

Viravathana and Marr^44^ calculated the vdW potential acting between two TiO_2_-SiO_2_ core-shell particles by splitting the potential between regions, such as between cores and shells.

We used refractive indices of 2.4, 1.43, and 1.33 for TiO_2_, SiO_2_, and water, respectively, in calculating the Hamaker constant *A* by Lifshitz theory (Eqs. (6) and (7)). The refractive index of TiO_2_ was determined by analyzing the interference of a thin film of synthesized TiO_2_ core particles adsorbed as two-dimensional colloidal crystals on a glass substrate. Other values are from literature^43^. For dielectric constants, literature values of anatase *ε* = 48 were used for TiO_2_, and 3.8 and 78.3 for SiO_2_ and water. *A* = 49.4 *k*_B_*T*, 1.27 *k*_B_*T*, and 5.77 *k*_B_*T* between TiO_2_ and TiO_2_, SiO_2_ and SiO_2_, and SiO_2_ and TiO_2_, respectively, in pure water at 25 °C. *A* between TiO_2_-TiO_2_ was about 40 times the value between SiO_2_-SiO_2_, and the vdW force became stronger when the shell was thinner.

### Interaction potential

The interaction pair potential was calculated as the sum of the Yukawa and vdW potentials. An example of a potential curve is shown in Fig. 9b. When the contribution of the electrostatic repulsive force expressed by the Yukawa potential is sufficiently significant, the total potential has a maximum. The value of the potential maximum (*U*_max_) at the maximum was calculated for various shell thicknesses. The results are shown in Fig. 9c.

The higher the salt concentration, the weaker the electrostatic interaction, so the potential maximum is lower. Also, the thinner the shell thickness, the stronger the vdW attraction, so the potential maximum is also lower. Thus, it is shown that the dissolution and desorption of the silica shell layer can cause particle aggregation due to the vdW force. Note that the concentration of free particles which are not involved in aggregation can be calculated as a function of time from the analysis of the rate of aggregation based on the Smoluchowski theory^45^. However, it is assumed that the particles irreversibly aggregate when they come into contact. Under the space experiment conditions (particle diameter 1 μm, particle concentration 0.1 vol%), the half-life *t*^1/2^ of the free particle concentration is *t*^1/2^ = 85 seconds when there is no interaction between particles, and 11 h and 69 days for *U*_max_/*k*_B_*T* = 10 and 15, respectively. Figure 9c shows the conditions for *U*_max_/*k*_B_*T* = 10 with red lines; the storage period at the ISS is about 250 days, and aggregation is expected within the storage period when the shell layer is thin, and the salt concentration is sufficiently high. However, the ionic strength expected from the conductivity measurements was about 100 µM, insufficient for the aggregation of most particles. Although the eluted silica layer was slight and the overall charge of the particles remained positive, it is possible that the silica layer eluted non-uniformly, resulting in non-uniform charge and aggregation.

### Implications of the present space experiments

In this study, associations of positively and negatively charged colloidal particles were conducted in aqueous media in the microgravity environment of the ISS. The obtained samples were immobilized in a polymer gel and analyzed by microscopic observation and small-angle and ultra-small-angle neutron scattering experiments on the ground.

Even for polystyrene particles with specific gravity *ρ* = 1.05, which is close to the specific gravity of the medium, the clusters’ average number in the space sample was ~50% larger than that of the ground control. The space sample also exhibited better structural symmetry than the ground sample for aggregates with association numbers of 4 and 3, as determined by image processing.

The present experiment enabled a detailed analysis of the aggregation structure of the high specific gravity (~3) titania particles, settling and forming macroscopic aggregates on the ground. Although not initially expected, during the waiting period on the space station (about 8 months), the silica coating layer on the titania particle surface degraded over time. Therefore, most of the particles became relatively large aggregates consisting of about 100–1000 particles, and only a few isolated clusters were formed. However, it was found that the dendritic aggregates with a fractal dimension of *D*_f_ ~ 2 were formed in space, while *D*_f_ was ~3 on the ground due to the compact aggregation of particles. The electrostatic interactions’ effect on clustering was also confirmed for titania particles.

Furthermore, titania particle tetrahedral clusters were obtained for the first time in space: tetrahedral aggregates were the building blocks of a diamond lattice, and the diamond structure of titania particles with a high refractive index acted as a photonic crystal with light-confining ability. Optical characterization of the space samples is planned in the future.

This study suggests that slight settling and convection effects on the ground can affect the aggregation of colloidal particles on the micrometer order. The findings obtained from these space experiments are expected to be useful for developing models used in structural materials design, including photonics.

## Methods

### Colloidal particles and characterization

Details of the synthesis of PS and titania particles are given in Supplementary Note 2. We summarized the characteristics of the particles used in this study in Tables 1a and 1b. Particle size was measured by scanning electron microscopy (JCM-6000, JEOL, Tokyo, Japan). The electrophoretic mobility of the particles was measured by a microscopic electrophoresis apparatus (type Zeecom ZC-3000U, Microtec Co., Ltd., Chiba, Japan) with more than 100 particles at [NaCl] = 10 μM. The zeta potential *ζ* of the particles was calculated from the mobility using Henry’s formula.

### Ground control experiment

PS#1 ([NaCl] = 0 μM) samples were taken out of the sample bag without being gelled. The electrical conductivity of the positively and negatively charged particle dispersions and *ζ* value of the particles were measured to determine how the samples changed over time. For the positive and negative particle dispersion systems, the electrical conductivity *σ* = 3.90 μS/cm and 5.46 μS/cm, respectively; for the five μM HCl solution, *σ* = 2.7 μS/cm, indicating that *σ* increased by about 1~3 μS/cm. This value is likely due to contamination by ionic impurities during storage, corresponding to 10~30 μM for [NaCl]. Also, *ζ* = +23.9 mV and −47.3 mV for positively and negatively charged particles, respectively (measured at [NaCl] =10 μM).

At the time of sample preparation (November 2019), *ζ* values were +38.7 mV and −50.1 mV, respectively. The *ζ* of positively and negatively charged particles were reduced by about 38% and 5%, respectively. The surface charge numbers calculated from the *ζ* using the Poisson-Boltzmann equation were +6450 (when filled: +3690) and −13,100 (when filled: −14,200), respectively. The large rate of decrease in positive charge may be due to the fact that the charge of the amino group that gives the positive charge is affected by slight changes in pH.

For sample PS#2, the two solutions were mixed to form clusters as in the space experiment. Then the bags were put under pseudo microgravity by using a rotator (type MTR-107, As-One, Tokyo, Japan, rotation radius = 6 cm) for two days at 3 rpm. Gelation was performed using a UV-LED lamp. Cross sections of the gels were observed with an optical microscope, and the elastic modulus was measured with a rheometer. The elastic modulus was *E*_0_ = 24.5 ± 4.2 kPa. Samples PS#3 and #4 were prepared similarly.

The titania sample #23 ([NaCl] = 0 μM) was taken out of the bag and the *ζ* of the particles were measured. The p- and n- titania particles had *ζ* = +37.7 ± 6.5 mV and −30.4 ± 4.8 mV, while the initial values were *ζ* = +48.7 mV and −36.1 mV, respectively. The other samples were rotated using a clinostat (Portable Microgravity Simulator PMS-VIII, AES Co., Ltd.) at 10 rpm for two days and then immediately gelled using a UV-LED lamp.

### Microscope observations

An inverted fluorescence microscope (ECLIPSE, Ti-S, Nikon, Co., Ltd.) with an oil immersion objective (100×, Plan Flour, Nikon) was used. Movies of the sample were taken while moving the viewing field, and the number of clusters having various *m* values was counted. Approximately 1000 clusters were counted in the total for PS samples. For the titania sample, more than 50 clusters were counted because most of the particles formed large aggregates.

Large aggregates of titania samples were observed using a confocal laser scanning microscope (type C2, Nikon) and an all-in-one microscope (BZ-X800, KEYENCE, Osaka, Japan) equipped with an optical sectioning module (BZ-H4XF, KEYENCE, Osaka, Japan).

The particles’ surface structure and elemental analysis were performed using a transmission electron microscope (type S-4800, Hitachi, Tokyo) and a EDS/EDX detector (ULTIM MAX65, Oxford Instruments, Tokyo, Japan) at the Analysis Center, Nagoya City University.

### Monte Carlo simulation

We calculated the equilibrium configurations of various clusters using the Monte Carlo (MC) method^46,47^ with experimental parameters for PS samples to see if the abovementioned experimental results are theoretically valid. Details of the calculations are given in Supplementary Note 4. Since the vdW force is sufficiently smaller than the electrostatic interaction in the PS sample, we solely used Yukawa-type potentials, Eq. (1), for the calculation.

In Fig. 4d, a plot of <*m*> versus [NaCl] obtained from the MC simulation is presented. The distribution of *m* at each *C*_s_ is shown in Supplementary Fig. 10. Although the simulation gave <*m*> values several times larger than in the experiment, <*m*> increased on increasing [NaCl] and reached a constant value. This trend was qualitatively consistent with the experimental results in the low [NaCl] region. Although we assume that the particles are perfectly smooth spheres in the MC simulation, actual particle surfaces are more or less rough. Consequently, when two particles come into contact, frictional forces are generated, preventing the particles’ most stable configuration. The observed difference in <*m*> between simulation and experiment appears, at least partly, to be due to the presence of the frictional forces.

The size distribution of actual particles (coefficient of variation = 4%) may also affect the <*m*> value. However, in MC simulations for particles with a size polydispersity of 4%, the *m* values at [NaCl] = 2 µM–100 µM agreed within 2% for the monodisperse case. Results are presented in Supplementary Fig. 11.

### Neutron scattering experiments

The neutron scattering experiments were performed by using a small-angle neutron scattering instrument (Quokka) and an ultra-small-angle neutron scattering instrument equipped with a Bonse-Hart camera (Kookaburra) at the Australian Centre for Neutron Scattering, Australian Nuclear Science and Technology Organisation (ANSTO). Combining these instruments evaluated microstructures from 1 nm to 10 µm. The medium of the experimental space samples was replaced from water with heavy water, and measurements were performed by increasing the difference in scattering length density between the polystyrene and titania particles and the medium. Gel matrix itself has negligible scattering (data are not included here) and was subtracted from overall scattering as background.

Particle association in gel matrix was studied using the Quokka small-angle neutron scattering (SANS) (ANSTO, Sydney, Australia)^48^ and Kookaburra ultra-small-angle neutron scattering (USANS) (ANSTO, Sydney, Australia)^49^ instrument at ANSTO. The gels were socked in D_2_O and then were loaded in the demountable sample holders (20 mm diameter for SANS and 40 mm diameter for USANS) with a 1 mm path length filled with D_2_O as a background medium. The scattering vector *q* is defined as *q* = (4πsin*θ*)/*λ*, where *λ* is the neutron wavelength and 2*θ* is the scattering angle. The SANS data were collected in the *q* range of 0.0007–0.1 Å^−1^, with source aperture to sample aperture distances of 12, 12, and 20 m-lens and sample to detector distance of 1.3, 12, and 20 m, and a neutron wavelength (λ) of 5.0 Å and 8.1 Å (for 20 m-lens optics), respectively. The USANS data were collected in the q range of 0.00004–0.001 Å^−1^, with *λ* of 4.74 Å. All the obtained SANS and USANS data were processed (data reduction, desmearing (for USANS data), and background subtraction) and combined using IGOR software. Data were then analysed further using in the software SasView5.

### Supplementary information

Supplementary Information

## Data Availability

The datasets generated and analyzed during the current study are available from the corresponding author upon reasonable request.
