# Author Correction: Clustering of charged colloidal particles in the microgravity environment of space

**DOI:** 10.1038/s41526-023-00313-z

**Published:** 2023-08-11

**Authors:** Hiroyuki Miki, Teruyoshi Ishigami, Junpei Yamanaka, Tohru Okuzono, Akiko Toyotama, Jitendra Mata, Honoka Komazawa, Yushi Takeda, Madoka Minami, Minori Fujita, Maho Doi, Tsunehiko Higuchi, Hiroshi Takase, Satoshi Adachi, Tetsuya Sakashita, Taro Shimaoka, Masae Nagai, Yuki Watanabe, Seijiro Fukuyama

**Affiliations:** 1 Graduate School of Pharmaceutical Sciences, Nagoya City University, 3-1 Tanabe, Mizuho, Nagoya, Japan; 2 Australian Centre for Neutron Scattering (ACNS), Australian Nuclear Science and Technology Organisation (ANSTO), Lucas Heights, NSW 2234 Australia; 3 Faculty of Pharmaceutical Sciences, Nagoya City University, 3-1 Tanabe, Mizuho, Nagoya, Japan; 4 Core Laboratory, Graduate School of Medical Sciences, Nagoya City University, 1 Kawasumi, Mizuho, Nagoya, Japan; 5 Japan Aerospace Exploration Agency (JAXA), 2-1-1 Sengen, Tsukuba, Japan; 6 Japan Space Forum (JSF), 3-2-1 Kandasurugadai, Chiyoda, Tokyo, Japan; 7 Advanced Engineering Services (AES) Co., Ltd., 1-6-1 Takezono, Tsukuba, Japan

**Keywords:** Colloids, Self-assembly

Correction to: *npj Microgravity* 10.1038/s41526-023-00280-5, published online 29 April 2023.

In page 2 the values were incorrectly stated as ‘0.1M dimethylacrylamide, 10 mM methylene-bis-acrylamide, 10 mg/mL VA-086’ where the correct value is ‘4.5v% dimethylacrylamide, 1.5 mg/mL methylene-bis-acrylamide, 0.1 mg/mL VA-086’.

In Table 1 of this article, the value in the column headed “Cv(%): of the row was affected”.

(1) p. 3, Table 1-a

(original)

**Table:** 

Particle	Cv	ζ
	(%)	(mV)
PS(+)	2.8	38.7
PS(-)	3.5	-50.1
TiO2(+)	3.4	48.7
TiO2(-)1	3.9	-56.2
TiO2(-)2	3.7	-50.4
TiO2(-)3	3.2	-44.5
TiO2(-)4	3.7	-36.1

(correction)

**Table:** 

Particle	*d*	*ζ*
	(nm)	(mV)
PS(+)	789 ± 22	38.7
PS(-)	1025 ± 36	-50.1
TiO_2_(+)	957 ± 33	48.7
TiO_2_(-)1	907 ± 35	-56.2
TiO_2_(-)2	1016 ± 38	-50.4
TiO_2_(-)3	1004 ± 32	-44.5
TiO_2_(-)4	1016 ± 38	-36.1

The original article has been corrected.

