# Supplementary material for: Clustering of charged colloidal particles in the microgravity environment of space
Source: NPJ Microgravity. 2023 Apr 29;9:33. doi: 10.1038/s41526-023-00280-5 (PMC10148850; doi:10.1038/s41526-023-00280-5)
Supplement: Supplementary file 1 — Supplementary Information [file 41526_2023_280_MOESM1_ESM.pdf]

Supplementary Notes

1. Space experiment

Colloidal samples were prepared on November 19, 2019, at the JAXA Tsukuba Space Center. Two samples having the same composition were prepared, one used for the space experiment and the other as the ground control sample. Then, the space samples were packed in a formed polystyrene storage container and sent to the launch site (Cape Canaveral Air Force Station, Florida, U.S.A.). The ground control samples were stored at JAXA Space Center at 4°C. On December 6, 2019, approximately 17 days after preparations, the samples were launched by a Falcon rocket (Space-X) equipped with a cargo spacecraft Dragon SpX-19 transport vehicle. On December 8, SpX-19 docked to the ISS and began storing samples at cabin air temperature. After SpX-19 was undocked, the samples were stored at cabin temperature for about one month and then in a refrigerator FROST on the ISS, maintained at 3-3.5°C.

The space experiment started on July 27, 2020, approximately eight months after the sample arrived at the ISS. The ISS crew removed the sample bags from the storage container, and the crew broke the separator between the two sample rooms by crushing the bags. Next, both bags were pushed 100 times alternately to stir and mix the colloid samples. Then the sample bags were attached to the experimental apparatus equipped with UV-LEDs, in the multi-purpose rack in the JEM of the ISS. The samples were kept in a microgravity environment. After two days (July 30), the samples were illuminated by UV-LED for two hours for gel immobilization.

The sample bags were stored in containers for collection and stored in the onboard storage room. They were returned to the ground by SpX-21 in January 2020. The samples were then delivered to the Nagoya City University laboratory in March 2021, stored in a refrigerator maintained at 4°C, and began analysis.

Each storage container is equipped with two temperature data loggers that automatically measured the internal temperature every 32 minutes, with one logging data from 0 to 182 days and the other from 179 to 361 days. The temperature history of the sample containers for the space experiment (averaged over three containers) is shown in Supplementary Figure 1. Although there are some differences in the storage temperature histories of space and ground samples, we assume that the degree of degradation of the ground and space samples is comparable, as will be discussed in Supplementary Note 6.

The effective acceleration of gravity on the ISS has been maintained at approximately 3×10^{-5} G. However, during the clustering experiment, the ISS was re-boosted for about 3 hours. At this time, the maximum effective acceleration of gravity was 1.7×10^{-4} G. The effective G during the re-boost is shown in Supplementary Figure 2. The average distance traveled by particles during the re-boost was estimated to be about 3 μm. We suppose that the re-boost did not significantly affect the results of this

experiment for the following reasons.

The sample container is made of the plastic film bag shown in Figure 2 of the main text. Under microgravity, the container bag is considered to have a shape that balances the internal pressure of the sample liquid and the force due to the elasticity of the plastic film of the sample bag. Since the effective G at the re-boost was sufficiently small compared to the internal pressure of the liquid, the deformation of the container is considered negligible. Also, the sample is sealed in a bag and has no free surface. For these reasons, we assume that the flow due to G is negligible.

Without flow inside the sample, all particles are subjected to a force in the direction of G and should move accordingly. Supplementary Figure 3 illustrates the effect of sedimentation. Although the travel distance ~3 microns is indeed larger than the particle diameter (1 micron), we believe that the re-boosting effect is negligible except near the sample bag walls if all the particles in the sample move in the same direction.

The portion of the space sample near the sample bag wall remained liquid in many cases, due to insufficient gelation. In addition, microscopic observations were made by cutting the sample with a scalpel and observing the cross section. Thus, since the portion of three microns from the sample bag surface was excluded from the evaluation, we assume that the effect of sedimentation by the re-boost was considered negligible in practice.

2. Synthesis of colloidal particles

Synthesis of positively charged polystyrene particles

Positively charged PS particles were synthesized by dispersion polymerization[2,3] as follows: 10.0 g of polyvinylpyrrolidone (PVP K30, Wako Pure Chemicals, Tokyo, Japan) was dissolved in 126 mL of ethanol and 14 mL of Milli-Q water with stirring and bubbled with N_2 gas. To this reaction solution, the solution was added 0.136 g of 2,2'-azobis(isobutyronitrile) (AIBN, Wako), 0.2 g of the cationic monomer 4-vinyl benzyl trimethylammonium chloride, 0.2 g of the red fluorescent dye Nile Red (Wako), 10 mL of the monomer styrene, and 0.5 mL of the divinylbenzene (Wako) were added in this order. The mixture was stirred in an oil bath at 70 °C for 24 hours. We determined the particle diameter by using a scanning electron microscopy.

Synthesis of titania particles

A partially modified sol-gel method for synthesizing titania particles using titanium tetraisopropoxide as starting material by Tanaka et al.[4]. A mixture of 2100 mL of methanol and 900 mL of acetonitrile was used as the solvent. To this solution were added 4 mL of Milli-Q water, 22.4 g

of dodecylamine (Wako), 4 mL of Tween 20 (Wako), and 20 mL of titanium tetraisopropoxide (TTIP, Wako) in this order, and the mixture was stirred at 10° C for 24 hours. Impurities were removed from the reaction solution by centrifugation and purified by dialysis. The medium was then replaced with methanol and dried dropwise on NaCl powder. The dried powder was calcined in a crucible at 400 ºC for four hours. The calcined solid was taken in a centrifuge tube and dispersed in Milli-Q water, and NaCl was removed by centrifugation to obtain an aqueous dispersion of titania particles. Wide-angle X-ray scattering confirmed that anatase-type titania crystals were obtained. SEM (type JCM-6000, JEOL, Tokyo, Japan) measurement evaluated the particle radius ap. The settling velocity v was measured in water, and the specific gravity ρ was determined from the values of a_p and v, assuming Stokes' law. For the titania particles used in the space experiment, ρ = 2.9±0.1.

Introduction of fluorescent dyes

Fluorescent dyes were introduced to the particles using the method of Van Blaaderen and Vrij[5]. For the positively charged particles, 2.5 mg of Rhodamine B-isothiocyanate (RITC, Sigma Aldrich, Missouri, U.S.A.) was dissolved in 2 mL of ethanol, to which 10 μL of 3-aminopropyltriethoxysilane (APTES, Shin-Etsu Chemical, Tokyo, Japan) was added in tiny drops. The mixture was then stirred for 24 hours. in a light-shielded condition. The compound (RITC-APTES) was obtained by adding RITC to the amino group APTES. Next, 13 mL of ethanol, 4.3 mL of Milli-Q water, 430 μL of tetraethoxysilane (Shin-Etsu Chemical), and 100 μL of RITC-APTES solution were added to a Teflon vessel and stirred. To this was added 620 μL of 28% ammonia water and 10 mL of titania particle dispersion (5 vol%, ethanol dispersion) and went for 24 hours. After the removal of unreacted material by centrifugation, the particles were dispersed in 30 mL of ethanol. Fluorescein-4-isothiocyanate (FITC, Wako) was introduced.

Modification by polyelectrolytes

To synthesize titania p-particles, we modified the particle surfaces with polyethyleneimine. 13 mL of ethanol and 4.3 mL of Milli-Q water were mixed as a solvent. 1.5 mL of a 50% solution of trimethoxysilylpropyl modified polyethyleneimine (solvent isopropanol, Gelest, Pennsylvania, U.S.A.) and 28% 620 μL of ammonia water were added and stirred, followed by the addition of 10 mL of titania particle dispersion with RITC and stirred at room temperature for 24 hours. The reaction solution was centrifuged to remove impurities, and the particles were dispersed in Milli-Q water.

Titania n-particles were prepared by modifying the particle surfaces with sodium poly (styrene sulfonate). First, vinyl groups were introduced onto the particle surfaces as follows. 30 mL of Fluorescein-loaded titania particle dispersion, 350 μL of γ-methacryloxypropyltrimethoxysilane (TPM, Shin-Etsu Chemical), and 850 μL of 28% ammonia water were added to a 200-mL flask and stirred for three hours. Then 20 mL of ethanol was added, and the ammonia was removed using an

evaporator at 55~60 ºC. The sample was redispersed in 30 mL of 70% EG after removing unreacted material by centrifugation. Poly(styrene sulfonate) sodium salt was then introduced by the following method: 1.0 g of styrenesulfonate sodium salt (NaSS) was dissolved in 60 mL of 70% EG, 30 mL of the above vinyl group introduced silica particle dispersion was added and bubbled with N_2 gas for 15 minutes. Next, 35 mg of 2,2'-Azobis[2-methyl-*N*-(2-hydroxyethyl)propionamide], a radical polymerization initiator, was dissolved in 10 mL of 70% EG, bubbled with N_2 gas for 10 min, and the entire amount was dropped into the reaction solution and stirred overnight. The product was purified by centrifugation and dispersed in Milli-Q water.

3. Gelation reagents

We have previously the immobilization of charged colloidal crystals using acrylamide and *N*-methylolacrylamide gel (NMAM) polymer gels[6]. However, the clustering of positive and negative particles was inhibited by the presence of NMAM, presumably due to slight hydrolysis of the NMAM molecules in the gelator solution, resulting in ionic impurities, so we choose to use dimethyl acrylamide (DMA) as a gel monomer that is less susceptible to hydrolysis.

4. Numerical simulation by Monte Carlo method

Monte Carlo simulations of the clustering were carried out to calculate the distribution of the number of associations. The calculated results agree with the results of Brownian dynamics simulations (see reference 7 for more details).

Simulation method

Numerical simulations have been carried out for a system of charged colloidal particles interacting with the Yukawa-type potential by applying the Monte Carlo method for canonical ensemble and using the Metropolis algorithm. Assuming the ergodic nature of the system, the steady-state distribution of particle configurations ω_s as,

$$\omega_s = \frac{1}{Q_N} \exp(-\beta U_s)\,, \tag{1}$$

and the Yukawa-type potential u_{ij} acting between particle i and particle j, using the thermal energy $k_B T$, the Bjerrum length l_B, and the Debye shielding length κ^{-1}, is as follows

$$u_{ij} = k_B T l_B \widetilde{Z}_i \widetilde{Z}_j \frac{\exp(-\kappa|r_i - r_j|)}{|r_i - r_j|} \quad \left(\widetilde{Z}_i \equiv Z_i \frac{\exp(\kappa a_i)}{1+\kappa a_i}\right), \tag{2}$$

where k_B is the Boltzmann constant, Bjerrum length $l_B = \frac{e^2}{4\pi\varepsilon k_B T}$ (e is the elementary charge), and

ε is the dielectric constant of the solvent. The Debye length κ^{-1} is defined by $\kappa^2 = 4\pi l_\mathrm{B} C$ using the total ionic concentration C in solution. The total potential energy U_s at state s is $U_s = \sum_{i<j} u_{ij}$ if it is approximated as the sum of pair potentials. In equation (1), Q_N is the coordination integral, which under constant volume and temperature is given as

$$Q_N = \int \exp(-\beta U_s)\, d\boldsymbol{r}_s^N, \tag{3}$$

where $d\boldsymbol{r}_s^N = d\boldsymbol{r}_1 d\boldsymbol{r}_2 \cdots d\boldsymbol{r}_N$ is the volume element concerning the position $\{\boldsymbol{r}_1, \boldsymbol{r}_2, \dots, \boldsymbol{r}_N\}_s$ in state s of N particles in the system. Also, if we define the transition probability from state t to state s as P_{ts}, the steady-state distribution of particle configurations from Eq.(1) as

$$\omega_s = \sum_t \omega_t P_{ts}. \tag{4}$$

In equilibrium, the transition probability is expressed by the following detailed balance, as

$$\exp(-\beta U_s) P_{st} = \exp(-\beta U_t) P_{ts}\ , \tag{5}$$

otherwise

$$\frac{P_{ts}}{P_{st}} = \frac{\exp(-\beta U_s)}{\exp(-\beta U_t)} = \exp(-\beta[U_s - U_t]), \tag{6}$$

is satisfied. The Metropolis algorithm below achieves thermal equilibrium conditions in the system by sampling particle configurations to satisfy this detail balance.

Metropolis algorithm

In a Monte Carlo simulation using the Metropolis method, the transition probability is determined by the energy difference between state t and state s. If $U_s < U_t$, the system transitions from state t to state s with probability 1; if $U_s > U_t$, the system transitions from state t to state s with probability $\exp(-\beta[U_s - U_t])$ (< 1). The specific method is as follows: for the nth time, let the system be in state t. Select one particle of the system at random, and let its particle number be α and its position vector is $\boldsymbol{r}_\alpha = (x_\alpha, y_\alpha, z_\alpha)$.

(a) The displacement vector $\Delta\boldsymbol{r}_\alpha = (\Delta x_\alpha, \Delta y_\alpha, \Delta z_\alpha)$ is prepared and the particle α is moved from $\boldsymbol{r}_\alpha$ to $\boldsymbol{r}_\alpha + \Delta\boldsymbol{r}_\alpha$. In this study, we use uniform random numbers ξ_x, ξ_y, and ξ_z in the interval (0,1],

$$\Delta x_\alpha = (1 - 2\xi_x)\delta, \quad \Delta y_\alpha = (1 - 2\xi_y)\delta, \quad \Delta z_\alpha = (1 - 2\xi_z)\delta. \tag{7}$$

In this study, $\delta = 1$ (i.e., δ is equal to the particle radius).

(b) Let state s be the system after the move. Calculate the potential energy $\Delta U = U_s - U_t$ of state t and state s, respectively. The value of the cut length R_c in the energy calculation is $R_c = 3l_p$. However, $l_p \equiv (V/N)^{\frac{1}{3}}$ is the average interparticle distance.

(c) If $\Delta U < 0$, the transition probability is 1. Therefore, state s is the new state t.

(d) If $\Delta U > 0$, the transition probability is $\exp(-\beta[U_s - U_t]) = \exp(-\beta\Delta U)$. The uniform random number in the interval (0,1] is used to determine whether to make the transition or not according to the probability. If the transition is made, state *s* is made into a new state *t* as before. If the transition is not made, state *s* is rejected.

(e) The state *t* determined in this way is the *n*+1st time, and the process starts again from (a). These operations are repeated until the system converges to an equilibrium state.

Numerical simulation conditions

We mainly used the conditions listed in Supplementary Table 1 as parameters for our simulations. These conditions are based on the conditions in the experimental systems conducted in our laboratory.

Supplementary Table 1

Positively charged particle		Negatively charged particle		Whole sample	
Diameter (nm)	Charge number	Diameter (nm)	Charge number	Particle's Volume fraction	Particle number ratio(+:-)
789	+9100	1000	-18000	0.0012	1:25

The equilibrium state was determined to have been reached when the total potential energy became constant. In this study, the distribution of the number of associations was obtained from the system judged to have reached equilibrium. Simulations were performed for a system with N_A=20 positively charged particles and N_B=500 positively charged particles. We used periodic boundaries as the boundary conditions of the system.

5. Structural Symmetry for Clusters of *m* = 2 and *m* = 3

The structural symmetry of tetrahedral clusters (*m*=4) of polystyrene particles is discussed in the main text（p.10）The bond orientation order parameter q_{tetra}, and we observed that the space sample has better symmetry. For clusters of *m*=3 and *m*=2, we defined q_3 and q_2, as in the case of tetrahedral clusters.

$$q_2 = 1 - \frac{1}{2}(\cos\theta + 1)^2, \tag{1}$$

$$q_3 = 1 - \frac{1}{3}\sum_{j=1}^{2}\sum_{k=j+1}^{3}\left(\cos\theta_{jk} + \frac{1}{2}\right)^2, \tag{2}$$

The q_3 and q_2 for polystyrene samples of various values of [NaCl] are shown in Figure 4. The distribution of q_3 and q_2 are presented in Figures 5 and 6 respectively. For q_3, as in the case of m=4 (q_{tetra}), the symmetry decreased with increasing [NaCl] for both space and ground samples. This indicates that electrostatic repulsion between attached particles contributes to the formation of clusters as in the case of m=4. Also, with the exception of [NaCl] = 50 mM, the space sample showed better symmetry than the ground sample, confirming the effect of microgravity, as in the case of m=4.

On the other hand, q_2 showed no clear dependence on [NaCl] for both space and ground samples, ranging from 0.6 to 0.8. This suggests that the contribution of electrostatic repulsive force to the aggregate formation is not significant due to the long distance between adhering particles. In such cases, the effect of microgravity is considered to be small.

The measure of symmetry, other than q_3 and q_2, is the sum of the bond vectors, $|\boldsymbol{r}_3|$and $|\boldsymbol{r}_2|$; regardless of m, if the generated cluster is perfectly symmetric, the sum of the bond vectors takes the minimum value of zero, and the better the symmetry, the smaller its value. Figure 7 shows the $|\boldsymbol{r}_3|$and $|\boldsymbol{r}_2|$ for space and ground samples, respectively. The same conclusions regarding the effects of [NaCl] and microgravity on the structural symmetry of the clusters were obtained using as when using q_3 and q_2.

6. Degradation of titania particles

As shown in Scheme 1 of the main text, the titania particles were coated with a shell layer of silica. Analysis of the ground control samples revealed that the shell portion of the silica has dissolved or detached over time. The amount of silicate monomer dissolved in the medium of the space experimental sample (27 months after preparation) was determined to be about 25% of that contained in the shell layer. For the ground control sample (29 months after preparation), silica elution was similar at 16%. See the following subsection for the method. However, when observed with a scanning electron microscope (SEM) equipped with an elemental analyzer (EDS), Si and Ti were detected in all particles observed (more than 100), and it was confirmed that not all of the silica-coated layer was detached. An example of the elemental distribution mapping results is shown in Supplementary Figure 8. The zeta potentials (ζ) of the ground control particles decreased from ζ = +63.4 mV to 49.1 ± 8.5 mV for the p-particles and from ζ = -46.7 mV to -39.3 ± 6.2 mV for the n-particles. Supplementary Figure 9 shows the distribution of ζ potentials. It is assumed that the positively charged polyelectrolyte modifies the titania particle surface decreases as the silica leaches out, decreasing the number of charges. Because the surface of pure silica is slightly negatively charged

in water, the particles become negatively charged when the polyelectrolyte is significantly removed. However, as shown in Supplementary Figure 9, no charge reversal of the particles was observed.

Note that titania particles act as photocatalysts. Therefore, if the surface silica layer was partly lost and the titania core surface appeared, the UV irradiation during gel fixation may have decomposed the rhodamine and faded the color. The change in fluorescence color observed in the space experiment may also be explained by the desorption of the silica layer.

The leaching and desorption of the silica layer are expected to change the interactions between particles as follows. (1) The electrostatic repulsive force between p-p and n-n particles and the attractive electrostatic force between p-n particles decrease due to the reduced number of charges. Also, (2) since the magnitude of the vdW interaction between particles is approximately proportional to the refractive index difference between the material and the medium, the vdW force between particles increases as the silica layer, which has a lower refractive index than titania, becomes thinner. Potential calculations considering (1) and (2) suggest that the contribution of the vdW attraction force exceeds the electrostatic repulsion force and that the vdW force may cause aggregation between n-n particles and between p-p particles. The ground control sample also observed that particles with the same sign charge formed macroscopic aggregates.

However, from the potential calculations, it is concluded that for the vdW force to exceed the electrostatic repulsive force between the particles, a large portion of the silica coat layer of the particles must be desorbed and dissolved, which is not consistent with the amount of silica dissolved (about 25% of the total amount). The partial desorption and dissolution of the silica coat layer may have created patchy, negatively charged silica regions on the surface of the positively charged particles. In this case, even though the total charge of the particles is positive, the particles may attract each other because of attraction between local positive and negative charged regions.

Determination of the dissolved silica concentration

Cut gel pieces of the space experimental samples were immersed in Milli-Q water and shaken for two days to extract the silicate dissolved in the liquid inside the sample gels. The amount of silica concentration in the extract was determined using the molybdenum blue method[8]. In an acidic sulfuric acid solution, the silicate reacts with ammonium molybdate(VI) tetrahydrate ($(NH_4)_6Mo_7O_{24}$) to form a yellow heteropoly acid. This was reduced to form molybdenum blue, and the absorbance was measured at 810 nm in a spectrophotometer. The silicate concentration of the sample was determined after heating at 80°C for 1 hour in 1M NaOH to hydrolyze the polysilicon acid.

Supplementary Figures

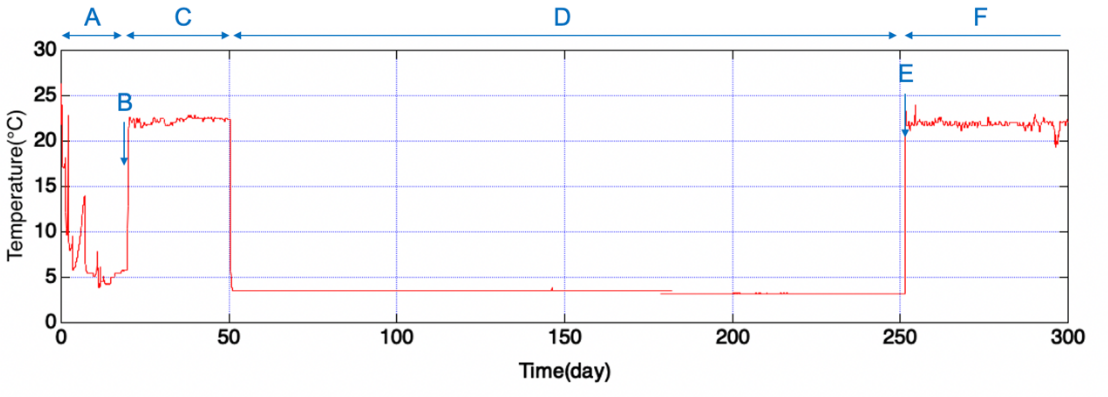

Supplementary Figure 1 Temperature history of the space experiment sample container. The horizontal axis shows the time (days) after sample filling, using two temperature data loggers, recording data from 0 to 182 days and from 179 to 361 days. A, Sample filled and transported from Tsukuba Space Center (Japan) to Kennedy Space Center (KSC, USA) for storage at the launch site; B, Launch; C, Dragon docks at ISS and stored at cabin temperature thereafter; D, Transferred to the ISS refrigerated storage (FROST) for storage; E, Experiment (cabin air temperature); F, Stored until sample retrieval.

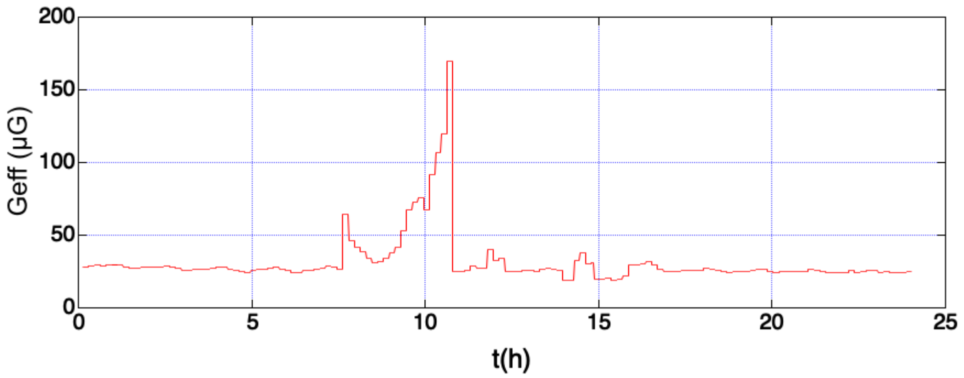

Supplementary Figure 2 Effective gravity acceleration G_{eff} of the ISS on July 27, 2020. Re-boost was performed from 7:38 to 10:47 (Greenwich Mean Time).

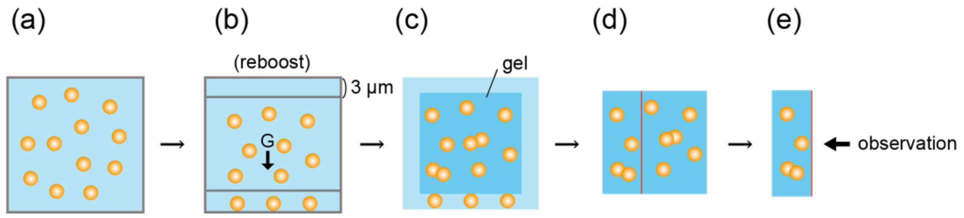

Supplementary Figure 3 Schematic illustration of the effect of re-boost. (a) uniformly dispersed particles, (b) sedimentation by re-boosting (assumed to settle uniformly 3 μm), (c) sample fixed in gel by UV irradiation at ISS after cluster formation. The area near the sample bag is not gelated. (d) Gel-fixed sample taken out from the bag after return to the ground. (e) Gel-fixed sample was cut and the cross section was observed under a microscope.

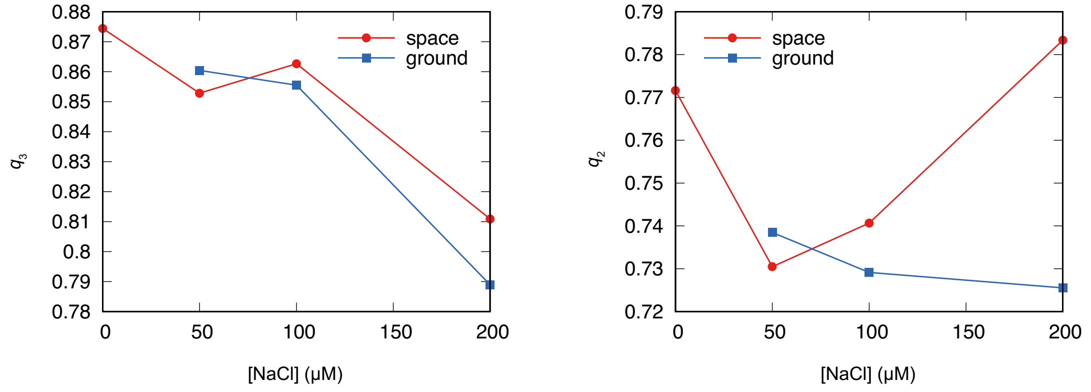

Supplementary Figure 4 Comparison of structural symmetry (q_3 and q_2) of m=3 and m=2 clusters in space and ground samples.

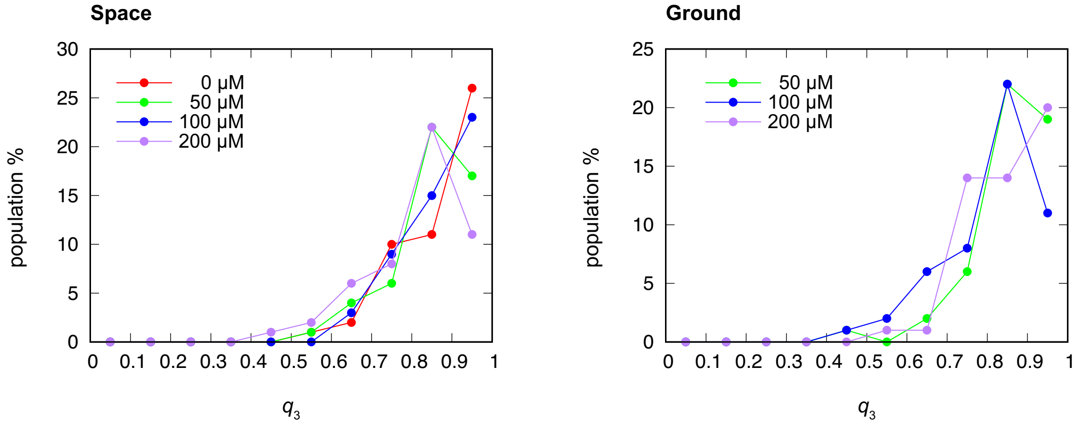

Supplementary Figure 5 Histograms of q_3 in space and ground samples.

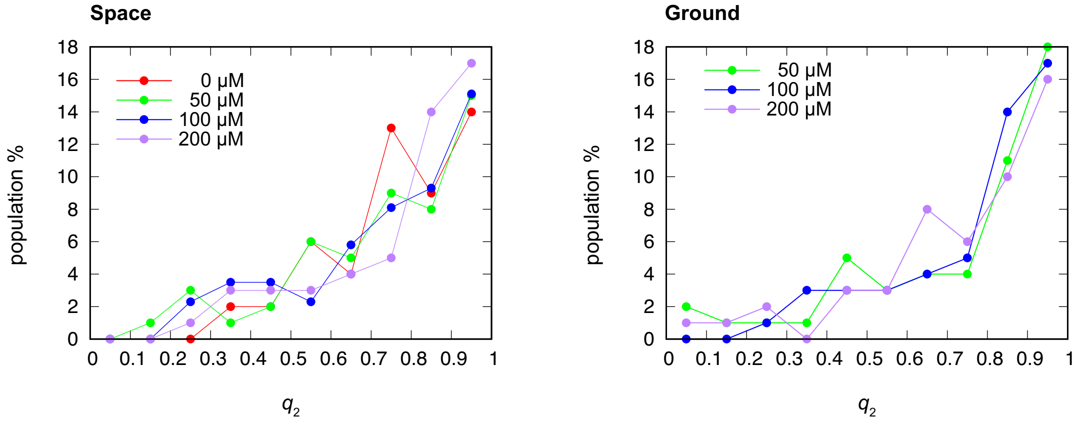

Supplementary Figure 6 Histograms of q_2 in space and ground samples.

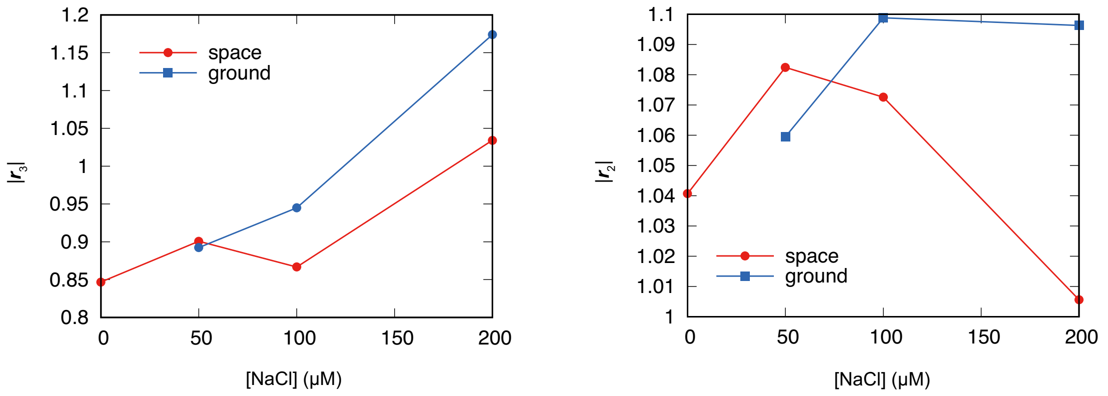

Supplementary Figure 7 Comparison of structural symmetry by using $|\boldsymbol{r}_3|$ and $|\boldsymbol{r}_2|$ in space and ground samples.

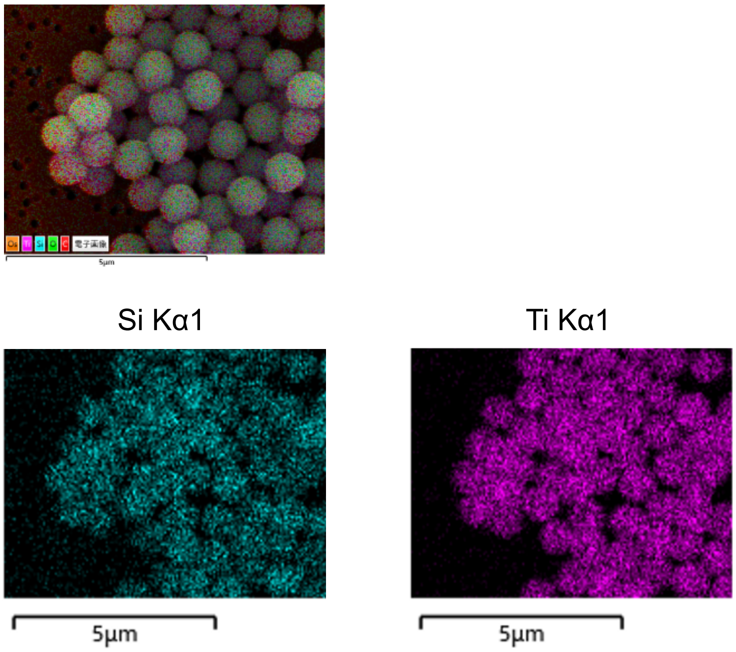

Supplementary Figure 8 EDS Mapping of TiO_2 particles (SiO_2 coated). Elemental analysis using SEM was performed on SiO_2-coated TiO_2 particles used in the space experiment because quantitative analysis of monosilic acid dissolved in the medium of the space sample indicated elution and desorption of SiO_2 due to degradation over time. Si and Ti were observed for all particles, indicating that not all Si coat was desorbed or dissolved.

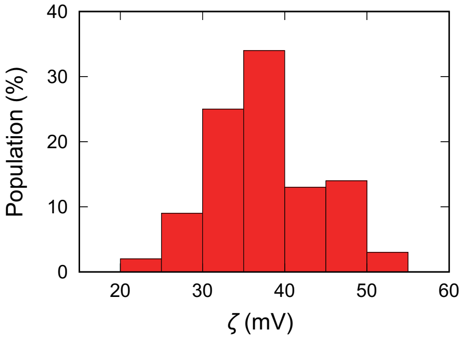

Supplementary Figure 9 An example of the distribution of ζ potential of ground-controlled TiO_2(+) (TiO_2#23) particles. The distribution of ζ potentials was examined by electrophoresis experiments on ground control samples of TiO_2 (+) particles that were not fixed in a gel. The average value of the potential of the space sample was lower than that of the ground control sample, but the charge did not invert to become negative.

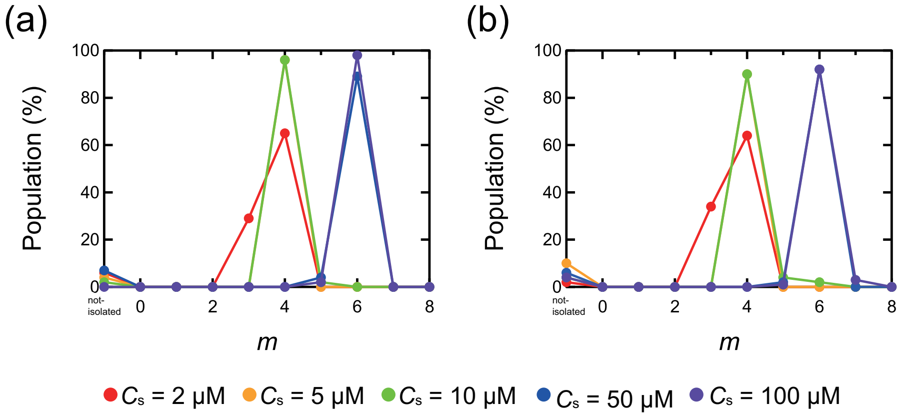

Supplementary Figure 10 The MC simulation on the effect of particle size distribution on the distribution of *m*. The distribution of *m* at various values of C_s for (a)monodisperse and (b) polydisperse (particle size distribution = 4% in standard deviation) polystyrene clusters. The size distribution was assumed to be Gaussian. In the simulations, the surface charge density was assumed as constant. The size polydispersity had a negligible effect on the distribution of *m*.

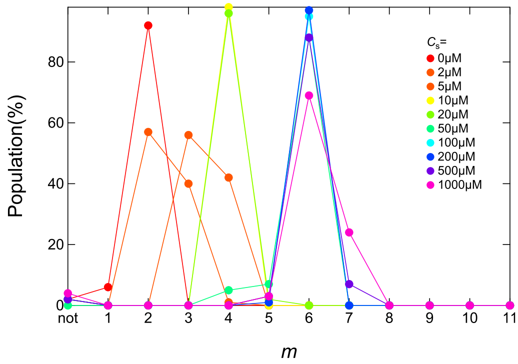

Supplementary Figure 11 Charge in a distribution of the association number *m* as a function of C_s (= 0, 2, 5, 10, 20, 50, 100, 200, and 500 μM). The cluster formation was judged from the radial distribution function.

References

1. Yamanaka, J. Okuzono, T., & Toyotama, A., Colloidal Crystals, Chapter 5, *Pattern Formation and Oscillatory Phenomena*, Kinoshita, S., ed., Elsevier, 2013.
2. Zhang, Q. et al. Preparation of fluorescent polystyrene microspheres by gradual solvent evaporation method. *Eur. Polym. J.* **45**, 550-556 (2009).
3. Wolters, J. R. et al. Depletion-Induced Encapsulation by Dumbbell-Shaped Patchy Colloids Stabilize Microspheres against Aggregation. *Langmuir* **33**, 3270−3280 (2017).
4. Tanaka, S. et al. synthesis of highly-monodisperse spherical titania particles with diameters in the submicron range. *J. Colloid Interf. Sci.* **334**, 188–194 (2009).
5. Van Blaaderen, A. & Vrij, A. synthesis and characterization of colloidal dispersions of fluorescent, monodisperse silica spheres. *Langmuir* **8**, 2921–2931 (1992).
6. Murai, M. et al. Unidirectional crystallization of charged colloidal silica due to the diffusion of a base. *Langmuir* **23**, 7510-7517 (2007).
7. Okuzono, T. et al. Numerical study of cluster formation in binary charged colloids. *Phys. Rev. E* **94**, 012609 (2016).
8. Volk, R. J. & Weintraub, R. L. Microdetermination of silicon in plants. *Anal. Chem.* **30**, 1011-1014 (1958).
